# Transport of Biologically Active Ultrashort Peptides Using POT and LAT Carriers

**DOI:** 10.3390/ijms23147733

**Published:** 2022-07-13

**Authors:** Vladimir Khavinson, Natalia Linkova, Ekaterina Kozhevnikova, Anastasiia Dyatlova, Mikhael Petukhov

**Affiliations:** 1 Department of Biogerontology, Saint Petersburg Institute of Bioregulation and Gerontology, 197110 Saint Petersburg, Russia; miayy@yandex.ru (N.L.); katena_94@list.ru (E.K.); nasya-nastasya@yandex.ru (A.D.); 2 Group of Peptide Regulation of Aging, Pavlov Institute of Physiology of Russian Academy of Sciences, 199034 Saint Petersburg, Russia; 3 The Laboratory “Problems of Aging”, Belgorod National Research University, 308015 Belgorod, Russia; 4 Petersburg Nuclear Physics Institute Named after B.P. Konstantinov, NRC “Kurchatov Institute”, 188300 Gatchina, Russia; michael.petukhov@gmail.com; 5 Peter the Great St. Petersburg Group of Biophysics, Higher Engineering and Technical School, Peter the Great St. Petersburg Polytechnic University, 195251 Saint Petersburg, Russia

**Keywords:** ultrashort peptides, POT, LAT, pathology, pharmacotherapy

## Abstract

Ultrashort peptides (USPs), consisting of 2–7 amino-acid residues, are a group of signaling molecules that regulate gene expression and protein synthesis under normal conditions in various diseases and ageing. USPs serve as a basis for the development of drugs with a targeted mechanism of action. The purpose of this review is to systematize the available data on USP transport involving POT and LAT transporters in various organs and tissues under normal, pathological and ageing conditions. The carriers of the POT family (PEPT1, PEPT2, PHT1, PHT2) transport predominantly di- and tripeptides into the cell. Methods of molecular modeling and physicochemistry have demonstrated the ability of LAT1 to transfer not only amino acids but also some di- and tripeptides into the cell and out of it. LAT1 and 2 are involved in the regulation of the antioxidant, endocrine, immune and nervous systems’ functions. Analysis of the above data allows us to conclude that, depending on their structure, di- and tripeptides can be transported into the cells of various tissues by POT and LAT transporters. This mechanism is likely to underlie the tissue specificity of peptides, their geroprotective action and effectiveness in the case of neuroimmunoendocrine system disorders.

## 1. Introduction

Peptides are molecules containing from 2 to 100 amino-acid residues linked by peptide bonds. According to the number of amino-acid residues, there is a division of peptides into polypeptides (from 10 to 100 amino-acid residues) and short peptides or oligopeptides (no more than 10 amino-acid residues) [1]. According to the classification of the International Union of Pure and Applied Chemistry (IUPAC), short peptides consist of 10–20 amino-acid residues, while polypeptides consist of 20 or more amino-acid residues [1,2]. According to another classification, short peptides include compounds up to 40 amino-acid residues in length [3]. In addition, there is a group of ultrashort peptides (USPs) consisting, according to some data, of 2–4 amino-acid residues [4], or, according to others, of 3–8 amino-acid residues [5,6,7].

Peptides possess antioxidant, antimicrobial, antibacterial, anti-inflammatory, anticarcinogenic, antitumor and immunoregulatory properties [8,9,10]. They regulate the functions of endocrine, nervous and immune systems and are involved in cell differentiation, apoptosis [11,12] and proliferation [11,13]. Thus, peptides have a wide range of targeted biological activities [14]. This allows us to consider them as promising molecules for the development of drugs [15].

The ability of positively charged short peptides to enter cells was first discovered in human immunodeficiency virus studies. Currently, these peptides are used to transport drugs into cells [16]. The use of fluorescence and electron microscopy combined with molecular modeling revealed passive transport of positively charged peptides into HeLa cells based on cell membrane fusion induced by transported peptides [17]. There is evidence in the literature that USP can enter into the cell with the participation of carriers of proton-dependent oligopeptide cotransporters (POT). Since ultrashort peptides have a short length (from 2 to 8 amino-acid residues), it can be assumed that their transport can be realized with the participation of L-type amino-acid transporters (LATs).

Di- and tripeptides, as well as various peptide-like compounds, such as β-lactam antibiotics, are absorbed into the epithelial cells of the intestine and kidneys using POT. These include PEPT1, PEPT2, PHT1, PHT2 carriers, consisting of 572–729 amino-acid residues (≈62–102 kDa). All the POT family members contain 12 transmembrane domains with N- and C-termini facing the cytosol. PHT1 and PHT2 recognize L-histidine as a substrate. PEPT1 and PEPT2 have high interspecies homology (about 80% in mice, rats, rabbits and humans), but sequence homology between these carriers for the same species is low (about 50%). Rat PHT1 and PHT2 have an amino-acid identity of about 50%, but they show insignificant sequence homology with PEPT1 and PEPT2 (less than 20%) [18].

PEPT1 and PEPT2 have a high degree of overlapping substrate specificity, being capable of amino-acid-sequence-independent transport of dipeptides and tripeptides [18,19,20,21]. It is unclear whether PHT1 and PHT2 proteins can transport the same range of di/tripeptides. However, the ability of PHT1/PHT2 to transport L-histidine distinguishes them from PEPT1/PEPT2 [19]. PEPT1 expression is more pronounced in the small intestine than in the kidneys. PEPT2 is predominantly expressed in the kidneys [22,23]. Both transporters are localized in the brush border membranes of epithelial cells. PEPT1 is a low-affinity peptide transporter, while PEPT2 is a high-affinity peptide transporter [18].

Amino-acid transporters are vital for nutrient uptake, neurotransmitter recycling, cell-redox balance and cell signaling. Essential amino acids are transported across the blood–brain barrier (BBB) using special carriers. Based on the difference in substrates, amino-acid carriers are divided into cationic, anionic and neutral.

LAT are heterodimeric amino-acid transporters. LAT1 predominantly transports neutral amino acids such as leucine, tryptophan, tyrosine and phenylalanine across the BBB via a Na-independent pathway. It is also involved in the brain delivery of some other biologically active substances, including L-DOPA, melphalan, gabapentin and baclofen [19]. LAT2 exhibits broader substrate specificity, transporting smaller neutral amino acids [20].

There are only a few publications describing the mechanisms of peptide transport into the cell, and they are mainly dedicated to peptides longer than 7–10 amino-acid residues. USP transport into the cell has not been properly studied yet. Based on literature data and analysis of the structure of active centers of amino acid and peptide carriers, it can be assumed that some USPs can interact with active centers of transporters, including LAT, and be transported into the cell. There are works devoted to some USPs that refute this assumption. However, no such analysis has been carried out for the entire pool of di- and tripeptides so far. It should also be noted that in the active centers of the transporters of peptides and amino acids there are areas with unclear function. Perhaps the biological meaning of such sites is the binding and transport of di- and tripeptides. In this regard, the purpose of the review is to analyze and systematize the possible mechanisms of cell USP transport.

## 2. POT Family Peptide Transporters (SCL15): PEPT1, PEPT2, PHT1, PHT2

The plasma membrane forms a natural barrier to amino acids, short peptides and other hydrophilic or charged substances. In order to maintain cellular homeostasis, a large number of membrane transporters for these molecules appeared during evolution.

Digestion of proteins in the intestinal lumen results in the release of individual amino acids and USPs. Transport of amino acids across the enterocyte plasma membrane occurs with the help of several amino-acid transporters [24], while transport of di- and tripeptides is carried out by the intestinal peptide transporter PEPT1. PEPT1 is a glycosylated protein containing 12 transmembrane domains. It is an H^+^-dependent peptide transporter that belongs to the POT membrane transporters family (SLC15). The POT family also includes PEPT2 isoform, synthesized in the cells of kidneys, lungs, brain and other tissues, as well as PHT1 and PHT2 transporters [25].

Oligopeptide transporters PEPT1 and PEPT2 provide proton-coupled active transport for a wide range of dipeptides and tripeptides, including zwitterionic, anionic and cationic peptides, as well as various peptide-like drugs (cefadroxil, enalapril and valaciclovir) [26].

Electrochemical gradient of protons across the membrane allows the uptake of di- and tripeptides against a concentration gradient. The function of PEPT1 depends on the function of the Na^+^/H^+^ exchanger NHE3, located on the apical membrane [27]. A decrease in Na^+^ concentration in cells serves as a driving force for NHE3, which removes protons from cells into the intestinal lumen. These protons are then returned to the cells along with the oligopeptides via the H^+^-peptide cotransporter PEPT1. After entering enterocytes, di- and tripeptides can be metabolized into amino acids or transported into the blood. In the intestinal cell culture, a stimulating effect of dipeptides on the cell level of PEPT1 was revealed. In vivo, the effect of dipeptides on PEPT1 synthesis may be mediated by its influence on gastrointestinal hormones. Insulin stimulates PEPT1 activity at the post-translational level by increasing PEPT1 incorporation into the plasma membrane [28].

PEPT2 can use di-, tri- and tetrapeptides as substrates (preferably dipeptides). Its affinity to substrates is significantly higher than that of PEPT1. In the kidneys, PEPT2 is localized on the apical surface of renal tubular cells, where it provides for the reabsorption of oligopeptides from urine [29].

According to the H^+^ gradient-dependent uptake mechanism, peptide transporters belong to “archaic” transporters. They are present in all living organisms and appeared in the early stages of evolution. Various isoforms of PEPT are present in the cell membranes of prokaryotes and simple eukaryotic cells. PEPT1-like form and renal PEPT2-like form can be found in worms (Caenorhabditis elegans) [30], fish (*Danio rerio*, *Gadus morhua*) [31,32], birds (*Gallus gallus domesticus*) [33] and mammals (*Oryctolagus cuniculus*, *Mus musculus*, *Homo sapiens*) [34,35].

Both PEPT isoforms have a broad substrate pattern that includes basically all di- and tripeptides derived from L-alpha amino acids, as well as a wide variety of derivatives, including drugs, such as beta-lactam antibiotics, angiotensin-converting enzyme inhibitors, protease inhibitors and antiviral drugs [36]. It was shown that PEPT1 can be used for many drug (zanamivir, oseltamivir, didanosine and others)-delivery purposes. It was supposed that PEPT1 can transport drugs into the bloodstream [37]. In the literature, it is mentioned that PEPT are critical facilitators of drug transport and distribution in the human body [38,39,40]. Brandsch M. et al. (2008) noted that concerning the interaction of well over 700 di- and tripeptides, amino acid with peptide transporters can be very important in creating drugs on the base of these short peptides [38]. It was supposed that the regulation of di/tri-peptide transport by POT plays an important role in physiological and pathophysiological mechanisms and drug creation [41].

Thus, PEPT transporters play an important role in the transport of various signaling molecules and drugs, including USP. In this regard, the study of the PEPT1-functioning mechanism and regulation of this process is of great importance for molecular medicine [42].

## 3. Localization of the POT Family Members in Various Organs and Tissues

The presence of peptide transporters in the brain has aroused interest concerning their physiological role and localization in the central nervous system’s (CNS) tissues and cells. In the mammalian CNS, various amino acids, amines and USPs function as neurotransmitters and neuromodulators. They also participate in the cellular metabolism and homeostasis regulation. PEPT2 has been found in the cerebral cortex, olfactory bulbs, basal ganglia, cerebellum and medulla oblongata [43]. PEPT2 is largely expressed in choroid plexus epithelial cells and ependymal cells. Its expression is observed exclusively on the apical (facing CSF) membrane of choroid plexus cells in newborn and adult rats, which suggests that PEPT2 is involved in the peptide transport from the CSF into the blood [44,45]. PEPT2 was also found to be expressed in the cerebral cortex differently depending on age; moreover, its levels in the fetus and newborn tissues were significantly higher than in adults. PEPT2 expression was found in neurons in adult and newborn rats, and in astrocytes of newborn rats. At the same time, PEPT2 expression in BBB endothelial cells was not detected, which was consistent with in situ hybridization studies [46] and the lack of evidence for the penetration of dipeptides into endothelial cells of brain microvessels [47]. No signs of PEPT1 expression in the brain were detected.

PEPT2 is expressed in epidermal keratinocytes [48]. The maximum concentration of PEPT2 is observed closer to the basal layer of the epidermis. This indicates that in epidermis, PEPT2 mediates the transport of oligopeptides from the basal layer [49]. PEPT2 mediates unilateral intracellular uptake of oligopeptides as an active transporter, activated by a transmembrane electrochemical proton gradient. Unlike keratinocytes, dermal fibroblasts and melanocytes do not express PEPT1 and PEPT2. The PEPT2 transporter, expressed mainly in keratinocytes, is assumed to be involved in the transcellular transport of oligopeptides and peptide-like drugs, delivering them to the underlying layers of the epidermis.

PEPT1 is a nonspecific transporter. It is localized mainly on the brush border membrane of the small intestine, and to a lesser extent in the kidney proximal tubules [29]. PEPT2 is a more selective transporter, and is expressed predominantly in the apical membrane of kidney cells. In the kidney proximal tubules, PEPT1 and PEPT2 are localized differently: PEPT1 is found in the S1 segment; PEPT2 is found in the S2 and S3 segments [50]. In rats, radioactively labeled molecular probes detected PEPT1 in the renal-cortex region, and PEPT2—in the outer part of the medulla [51]. With a knocked-out PEPT2 gene, a violation of peptide absorption in kidneys and choroid plexuses was revealed in mice [52,53]. PEPT1 and PEPT2 may be applicable in the pharmacokinetics of b-lactam antibiotics by transporting them to target cells and promoting reabsorption in renal tubular cells after glomerular filtration [18].

Relatively little is known about the expression and distribution of PHT1 and PHT2 in various animal and human tissues. PHT1 mRNA has been found in the choroid plexus cells of the brain and retina in rats. PHT2 transcripts were expressed mainly in the lymphatic system, lungs and spleen of rats, but were practically not detected in the brain [54,55]. SLC15A4 and SLC15A3 transcripts, encoding PHT1 and PHT2, have been found in human- and rat-intestinal-tissue segments. Moreover, immunohistochemical analysis has shown that PHT1 was expressed in the small intestine villous epithelium [56].

Thus, PEPT1 and PEPT2 transporters are localized in cells of various organs and tissues. PEPT1 is predominantly found in kidney and intestinal cells, whereas PEPT2 is detected in CNS neurons and vascular endothelium. These data are important in the context of understanding the tissue-specific action of some USPs. It can be assumed that di- and tripeptides, being transported into tissues, depending on their structure, are capable of interacting with PEPT1 and PEPT2, and thus can exhibit their protective properties against cells of various tissues.

## 4. Participation of the POT Family Transporters in Various Physiological Processes under Normal and Pathological Conditions

Due to their difference in tissue distribution and expression patterns, POTs are thought to have different functions in vivo. As the predominant (and probably the only) POT located on the small intestinal brush border membrane, PEPT1 is the transporter responsible for the absorption of small peptide fragments during protein digestion. It may also be the main carrier responsible for the absorption of peptidomimetics, such as some angiotensin-converting enzyme inhibitors and the antiviral drug valaciclovir.

It is suggested that based on the analysis of the structure of the PEPT2 transporter, it is possible to develop peptidomimetics for the treatment of lung pathology [57]. Peptidomimetics based on Au III-peptidodithiocarbamato complexes, transported by PEPT1 and PEPT2, suppressed the growth of human tumor cells (lung cancer, breast cancer, epidermoid carcinoma) in vitro [58]. In another study, peptidomimetics containing gold particles and transported by PEPT1 and PEPT2 also suppressed the growth of various human tumor cell lines [59].

Despite PEPT1 and PEPT2 expression in the nephron proximal tubule, recent in vivo studies have shown that PEPT1 plays a minor role in the reabsorption of dipeptides and aminocephalosporin from the tubular fluid. PEPT2 is the main transporter involved in the renal reabsorption of peptide substrates and peptidomimetics. PEPT2 localization on the apical membrane of choroid plexus epithelial cells contributes to the maintenance of neuropeptide homeostasis and the removal of neurotoxins from the brain. This localization also makes PEPT2 an attractive target for the peptidomimetics transport to the brain [18].

PEPT1 involvement in the pharmacokinetics of a large number of drugs causes interest in studying the regulation of PEPT1 under various physiological and pathological conditions [60,61].

Peptide transporters, PEPT1 in particular, are considered as drug-delivery systems [62]. Particular attention has been paid to the involvement of PEPT1 in the pathophysiology of gastrointestinal disorders, especially its role in inflammatory bowel disease (IBD). The H(^+^)/peptide transporter, PEPT1, is a key molecule promoting the development and progression of IBD [63,64]. Increased expression of PEPT1 in the colon during its inflammation contributes to the development of further inflammatory processes and carcinogenesis [65,66].

According to some data, PEPT1 can transport bacterial peptides into cells. This internalization promotes interaction between bacterial peptides and innate immune receptors, including NOD, thus activating the pro-inflammatory cascade. Future potential therapies for IBD may target inflammatory foci using PEPT1 ligands such as Lys-Pro-Val, chemically modified PEPT1-transported prodrugs or probiotics that downregulate PEPT1 expression in the small and large intestine [65].

Middle-aged animals revealed a higher expression of PEPT2 and an increased rate of β-Ala-Lys substrate uptake compared to young animals. These results support the idea that dipeptides may be effective myocardial protective agents in older animals; their increased uptake may be a manifestation of a compensatory mechanism in ageing [67].

In addition to the high intestine expression level, PEPT1 is also found in some tumors, such as pancreatic carcinoma, prostate cancer, gastric cancer, etc. [68,69]. PEPT1 can be specifically expressed in human hepatocellular carcinoma tissue and cell lines. It also possesses transport activity to deliver oligopeptides or peptidomimetic molecules. A possibility for a new therapeutic antitumor strategy, in which PEPT1 was considered as a target molecule that increases the antitumor efficacy of doxorubicin, was explored. Doxorubicin was conjugated with Gly-Gly-Gly tripeptide-ligand, which is a PEPT1 substrate. When using the doxorubicin-tripeptide conjugate for antitumor therapy of hepatocellular carcinoma, a decrease in the doxorubicin side effects was observed. It was suggested that PEPT1 might be a new target molecule for human hepatocellular carcinoma therapy [70].

The effect of acute stress or inflammation on PEPT1 function is not well described, despite the evidence that other membrane transporters’ expression may be inhibited during inflammation [71,72,73]. The influence of inflammation on the intestinal absorption of dipeptides has been established [74]. PEPT1 can activate inflammatory processes by facilitating intestinal absorption of peptides of bacterial origin. This effect can be attenuated by PEPT1 substrates in vitro [75]. PEPT1-mediated intestinal absorption is hypothesized to be preserved and potentially increased in inflammatory conditions, making PEPT1 a target for drug delivery for IBD therapy.

Regulation of the PEPT1 transporter synthesis in intestinal cells through dietary changes has been demonstrated in several studies [76,77,78]. Increasing the protein content enhanced the absorption of dipeptides in the small intestine of rats, as well as the levels of PEPT1 mRNA and protein. Moreover, the addition of selected amino acids or dipeptides to the culture medium increased dipeptide uptake, mRNA levels and PEPT1 protein levels. Analysis of PEPT1 promoter revealed a gene region that responded to all dipeptides studied, as well as to individual amino acids, such as Lys, Arg and especially Phe [79].

It has been established that starvation reduces the transport of Gly-Gln dipeptide localized in kidney cells [80,81].

In *Saccharomyces cerevisiae* yeast, gene expression of Ptr-2 peptide transporter [82] is regulated by dipeptides that activate the ubiquitin-dependent proteolytic pathway [83].

According to some data, PHT1/PHT2 transporters have not been detected in BBB cells [84,85]. However, another study suggested that they may participate in the intracellular USP transport [58]. There are data that PHT1/PHT2 is involved in the pathogenesis of irritable bowel syndrome, Crohn’s disease and ulcerative colitis [86].

Thus, POT family proteins are important for the transport of peptidomimetics, drugs and other signaling molecules in the kidneys, gastrointestinal tract and brain. The role of POT family transporters in the pathogenesis of diseases of the cardiovascular, immune and excretory systems and gastrointestinal tract has been described.

## 5. Role of PEPT1 and PEPT2 in the Transport of Ultrashort Peptides

The spectrum of USPs’ biological activity is wide: they regulate the functions of the endocrine, nervous and immune systems. The mechanism of action of peptides consists in their ability to regulate gene expression and protein synthesis in plants, microorganisms, insects, birds, rodents, primates and humans [10,14]. USP can penetrate cell nucleus and nucleolus and interact with the nucleosome, histone proteins and single- and double-stranded DNA. DNA–peptide interactions are vital for template synthesis reactions, including sequence recognition in gene promoters, replication, transcription and repair. USPs are able to regulate the DNA methylation status, which is an epigenetic mechanism of gene activation or repression in normal, pathological and ageing conditions [14]. In this regard, it can be assumed that evolutionarily USPs, similar to the POT family peptide carriers, were one of the first signaling molecules capable of cellular homeostasis regulation.

Violation of peptide bioregulation reduces the body’s resistance to external and internal destabilizing factors, which is one of the reasons for accelerated ageing and development of age-associated pathology [14]. In this regard, disruption of peptide transport may be an important link in the implementation of ageing mechanisms and pathogenesis of a wide range of diseases.

PEPT1 and PEPT2 carriers transport mainly USP. Irie M. et al. (2005) studied the transport of neutral and charged USP with the participation of PEPT1. Transfer of Gly-Sar (glycyl-sarcosine) dipeptide into the cell using PEPT1 was described by methods of physicochemistry and molecular modeling [87].

Transport mechanisms of antihypertensive tripeptides LKP (Leu-Lys-Pro) and IQW (Ile-Gln-Trp) obtained from egg white were studied using the system of co-cultivation of monolayers of Caco-2 and HT29 cell lines. The results showed that the transport was significantly inhibited by Gly-Pro dipeptide, a competitive substrate of the PEPT1 peptide transporter. Transport from the apical to the basolateral side was significantly higher than in the opposite direction. These results indicated that PEPT1 was involved in the transport of LKP and IQW peptides. Moreover, siRNA was also used to knock PEPT1 expression down, and inhibited the transport significantly, suggesting that PEPT1 was involved in the transport process. Thus, an active pathway via PEPT1 transporter for the antihypertensive peptides LKP and IQW lies through monolayers of co-cultured Caco-2 and HT29 cell lines [88].

In Xenopus laevus oocytes, a new function of the β-Klotho protein was revealed, which consisted in the activity reduction for peptide transporters PEPT1 and PEPT2 [89]. As shown for the TRPV5 epithelial Ca^2+^ channel [90] of amino-acid transporters [91], β-Klotho can stabilize transport proteins in the cell membrane and also suppress the action of peptide transporters. β-Klotho protein can probably affect the intestine transport of peptides. Peptide transport was activated in β-Klotho-deficient mice. It is assumed that an age-related decrease in the β-Klotho synthesis leads to a slowdown in peptide transport in intestinal cells [30].

The interaction of the antibacterial phosphonodipeptide alaphosphalin with mammalian H(^+^)/peptide cotransporters was studied in Caco-2 cells expressing low-affinity intestinal-type PEPT1 and SKPT cells expressing high-affinity renal-type PEPT2. Alafosfalin inhibited [(14)C]glycyl-sarcosine (Gly-Sar) uptake for PEPT1 and PEPT2, respectively. In both cell types, alaphosphalin was shown to affect only the affinity constant, but not the maximum uptake rate of glycylsarcosine (Gly-Sar). It has been established that alaphosphalin interacted with H(^+^)/peptide symporters and was transported through the intestinal epithelium in the H(^+^)-symport. Dipeptides with C-terminal carboxyl group substituted with a phosphonic function are high-affinity substrates for mammalian H(^+^)/peptide cotransporters. The antibacterial phosphonodipeptide alaphosphalin interacts with both mammalian H^+^/peptide symporters with high affinity. PEPT1 and PEPT2 do not seem to distinguish between a dipeptide and its derivative, where the C-terminal carboxyl group is replaced by a phosphonic acid. Phosphonodipeptides are of interest for studying the structural affinity bonds of PEPT1 and PEPT2 substrates [92].

There is evidence that PEPT1 may not transport all tripeptides into the cell [93]. The transepithelial transport of the VLPVPQK peptide (C peptide), which is an antioxidant and an angiotensin-converting enzyme inhibitor, via the PEPT1 transporter also remains poorly understood [94].

Thus, PEPT1 and PEPT2, as well as PHT1 and PHT2, belonging to the SLC transporters family, are the main peptide transporters in the body responsible for the proton-coupled transport of dipeptides and tripeptides. Their main function is to take up nitrogen in the small intestine (PEPT1) and reabsorb nitrogen from the glomerular filtrate in the proximal tubule of the kidney (PEPT1 and PEPT2). PHT1 and PHT2 remain the least studied vectors of this family, although their role in the pathogenesis of gastrointestinal disorders has been suggested [61,62].

There are some functional differences between PEPT1 and PEPT2. Firstly, localization: PEPT1 is mainly localized on the brush border of the small intestine and to a lesser extent in the renal epithelial cells, whereas PEPT2 is widely distributed in body tissues with the highest localization in renal epithelial cells. Secondly, PEPT1 is a low-affinity, high-capacity transporter, while PEPT2 is a high-affinity, low-capacity transporter. Although both transporters have a fairly wide substrate range, it is believed that the range of PEPT2 substrates is less than that of PEPT1. PEPT1 transporter is better characterized than PEPT2. This is partly due to the fact that PEPT1 is a target for prodrug delivery.

## 6. Amino-Acid Transporters LAT1 and LAT2: Possible Involvement in Peptide Transport

Among the amino-acid transport systems, L-amino-acid transporter (L system) is an important mechanism that ensures the transport of large neutral, branched or aromatic amino acids into cells. Originally the L system was identified in Ehrlich ascitic carcinoma cells [95]. LAT1 and LAT2 are the most studied carriers belonging to the SLC7 family. LAT3 belongs to SLC43 family. LAT4 was discovered later and shows 50% homology with LAT3 [96].

The LAT1 transporter consists of 507 amino-acid residues with a molecular mass of ~55 kDa [97]. No splicing variants of this protein have been described so far. Based on GeneBank data [98], LAT2 has two alternative transcripts: NM 012244.2 (11 exons) and NM 182728.1 (9 exons). The latter probably codes for a truncated protein containing 413 amino-acid residues (~46 kDa), while the former codes for a protein of 535 amino-acid residues (~58 kDa).

LAT1 and LAT2 are Na^+^ independent transporters [20]. They are amino-acid exchangers (antiporters) with a 1:1 stoichiometry—the transporter carries one amino acid out of the cell, while another amino-acid molecule is transported into the cell at the same time. LAT1 and LAT2 have similar but not identical selectivity for intracellular and extracellular substrates [75].

The function of LAT1 and LAT2 is most likely to balance the distribution of amino acids between the two membranes, while other transporters determine only the forward flow of amino acids. The transport activity of LAT1 and LAT2 has different sensitivity to extracellular pH [99]. Extracellular pH does not affect LAT1, while the LAT2-stimulated uptake is higher at acidic than at neutral pH. LAT1 forms a heterodimeric complex with the auxiliary protein 4F2hc and predominantly transports most of the essential amino acids: leucine, isoleucine, valine, phenylalanine, tyrosine, tryptophan, methionine and histidine [100,101]. LAT2 demonstrates a wider range of selectivity than LAT1, also transporting some smaller amino acids (glycine, alanine, serine, threonine, cysteine, glutamine, aspartate) [102], but in general LAT2 shows a lower substrate affinity than LAT1 [98].

LAT1 and LAT2 have been suggested to be involved in the transport of several drugs such as L-DOPA, melphalan, baclofen, 3-*O*-methyldopa, alpha-methyltyrosine, gabapentin, alphamethyldopa and thyroid hormones [103]. These proteins are involved in the transport of cysteine [104,105] and pregabalin [106] conjugates. LAT1 and LAT2 are localized in different tissues and their localization is species-specific. LAT1 is expressed in the brain, placenta and tumor tissues. It has been reported that LAT1 can be localized in the apical and basolateral membranes of BBB cells [107] and in the placental membrane (on the maternal side) [108]. Thus, LAT1 is involved in the transport of amino acids into proliferating cells and through some endothelial and epithelial barriers. LAT2 is expressed in the kidney, colon and intestine and is involved in the basolateral outflow of transepithelial amino-acid transport in the kidney and intestine [109,110]. However, a very low LAT2 mRNA expression in the human gut has been reported in one of the studies. Another study detected LAT1 expression in the colon of rats, as well as in some intestinal cell lines [111]. LAT2 presence in the BBB is controversial: low levels of LAT2 transcript have been reported in mice, rats and humans [93,104], but this finding was not supported by other studies [112,113].

Leucine transport via LAT1 in porcine kidney cells LLC-PK1 has been described [113]. LAT1 expression is regulated by the availability of amino acids. Interestingly, arginine, which is not a LAT1 substrate, has an effect on LAT1 transcription in liver cell lines [112,113]. LAT1 is elevated in hepatocytes during arginine deprivation. Such induction may also affect the absorption and distribution of drugs that are transported by LAT1 and LAT2. Pathological conditions such as hepatic encephalopathy or aminoaciduria can alter LAT1 expression in the BBB [114]. The regulation of LAT1 expression has been associated with aldosterone [115], arginine vasopressin and adrenergic agents. Moreover, LAT1 is highly expressed in many tumor cell lines, probably to provide essential amino acids to proliferating and dividing cells [101,116].

## 7. Participation of LAT1 and LAT2 in Various Physiological Processes during Ageing and Pathology

Uptake of aromatic amino acids, such as L-tryptophan, by activated lymphoid cells occurs primarily through the L1 systemic transporter, a heterodimer comprising the heavy chain of CD98 and the light chain of LAT1. Regulation of amino-acid transport through LAT1-CD98 heterodimer is associated with the activation and differentiation of T-cells [117]. CD69 has been found to associate with the LAT1-CD98 transport complex and enhance the uptake of L-tryptophan, a metabolic precursor of Ah receptor ligands, which promotes IL-22 secretion [118,119]. IL-22 induces keratinocyte proliferation via the PI3KAkt-mtorc37 signaling pathway. CD69 was associated with the LAT1-CD98 amino-acid transfer complex on the plasma membrane of activated T cells and controlled mTORC activation in skin cells during an autoimmune inflammatory response [117].

An elevated LAT1 expression is observed in keratinocytes and skin-infiltrating lymphocytes of psoriatic lesions in humans and mice. However, deletion of LAT1 in keratinocytes does not attenuate the inflammatory response or their proliferation, which might be supported by the increased expression of alternative amino-acid transporters LAT2 and LAT3 [120].

LAT1 expression is upregulated in a number of primary tumors and metastatic lesions from more than 20 tissues/organs [121]. Moreover, correlations between LAT1 expression and negative prognosis have been shown in various tumors, such as breast cancer [122], including its highly proliferative ER+ subtype [123], bladder cancer [124], lung adenocarcinoma, lung neuroendocrine tumor, adenocarcinoma pancreatic ducts [125] and biliary tract cancer [126]. Based on these data, LAT1 was considered as a molecular target for anticancer therapy. Several LAT1-selective inhibitors have been synthesized [127,128,129,130]. They have demonstrated pronounced antitumor effects in animal models [131,132].

LAT1 influence on the endothelial cell functions in tumors has not been elucidated. In a model of rat bladder carcinoma induced by N-butyl-N-(4-hydroxybutyl)nitrosamine, increased LAT1 expression in tumor-associated microvessels was recorded [133]. A clinical and pathological study of human glioma showed LAT1 expression in both vascular endothelial cells and tumor cells, demonstrating significant correlations of LAT1 expression with the degree of pathology and intratumoral density of microvessels [134]. A connection between LAT1-mediated amino-acid signaling and growth-factor-dependent pro-angiogenic signaling, converging on the nutrient-responsive concentrator kinase mTORC1 for angiogenesis regulation, has also been revealed. The study of LAT1 functions may be promising for the development of modern cancer treatment methods, antiangiogenic therapy in particular [135]. LAT1 is suggested to play a crucial role in tumor-associated metabolic networks, supplying tumor cells with essential amino acids [136].

Tyrosine transport in human fibroblasts has been characterized by L-transporter isoforms (LAT1, LAT2, LAT3, LAT4). This study showed that LAT1 is involved in 90% of the total tyrosine uptake, as well as 51% of alanine. Not more than 10% can be accounted for by LAT2, LAT3 and LAT4 isoforms. LAT2 appears to be weak in tyrosine uptake. Alanine inhibited tyrosine transport up to 60%. Transport of tyrosine through LAT1 isoform had a higher affinity compared to other L-system transporters. Thus, LAT1 isoform is the main tyrosine transporter in human fibroblasts. It has been shown that there is competition between tyrosine and alanine for transport via LAT1 and LAT2 isoforms [137]. LAT1 transporter is involved in the pathogenesis of neurodegenerative disorders, amyotrophic lateral sclerosis (ALS) in particular. LAT1 has been shown to be involved in the uptake of [14C]L-citrulline by motor neuron-like cells NSC-34. The level of LAT1 expression was lower in superoxide dismutase 1 (NSC-34/hSOD1^G93A^) mutant cells compared to the control. Similarly, in the spinal cord of ALS in transgenic mice, a decrease in LAT1 synthesis in the motor neurons of mice with ALS was found. However, an increase in LAT1 expression in nonmotor neurons and astrocytes was observed in the gray matter of the spinal cord of mice with ALS [138].

As mentioned above, LAT1 is responsible for the transport of large neutral L-amino acids, as well as several drugs and prodrugs, across the BBB. However, the BBB is not the only barrier preventing the effective action of drugs in the brain. Brain parenchymal cell membranes represent a secondary barrier to drugs with intracellular target sites. In a study by Huttunen et al. LAT1 expression and function were quantified in cultures of primary neurons, astrocytes and immortalized BV2 mouse microglia. LAT1 was detected in all three cell types. The most active transport involving this carrier was found in astrocytes. LAT1 provided transport of the prodrugs developed during the study into the cell. Interestingly, cellular uptake of prodrugs was higher in cells with the Alzheimer’s disease phenotype. Thus, facilitating the transport of drugs and their targeted delivery to brain cells can be achieved be means of LAT1 and prodrug creation [139].

LAT2 transporter has been shown to be expressed in the lens epithelium of mice. The deletion of LAT2 induced a sharp decrease in the level of essential amino acids in the lens. Interestingly, the absence of LAT2 resulted in an increased incidence of cataracts in mice, particularly in older females. Screening for LAT2 in patients diagnosed with congenital or age-related cataracts revealed one homozygous single nucleotide deletion that runs in a family with congenital cataracts. In a study on HeLa cells, mutant LAT2 did not provide amino-acid uptake. Heterozygous LAT2 variants have also been found in patients with cataracts [140].

Mice with LAT1 gene knockout did not survive the embryonic period [141]. In addition, inhibition of LAT1 reduced the viability of human umbilical vein endothelial cells (hUVEC), human primary aortic smooth muscle cells, and two human kidney cancer cell lines [142,143]. While LAT1 deficiency has been identified in many pathophysiological processes, it may also be relevant to pregnancy pathologies such as fetal-growth restriction [144,145]. However, a recent study found elevated LAT1 levels and fetal-growth restriction in patients with preexlampsia, which was interpreted by the authors as a compensatory mechanism [146]. This reinforces the idea that LAT1 can perform a regulatory function by supplying amino acids to mitigate concomitant oxidative stress [147]. The essential role of LAT1 in the human placenta is further demonstrated by its high cytotrophoblast expression [148]. Human trophoblasts have also been shown to express LAT1 to a greater extent than human placental endothelial cells. The role of LAT1 in placentation [149] and cytotrophoblast development [150] was demonstrated earlier.

Thus, dysfunction of LAT amino-acid transporters can result in embryogenesis disruption, pregnancy pathology, immunopathology, oncogenesis, development of neurodegenerative diseases and retinal pathology.

## 8. Possible Mechanisms for Ligand Transport by LAT1

At the first stage, to assess the possible role of LAT1 in the USP transport across the cytoplasmic membrane of the cell, a number of studies on the interaction of this transporter with amino acids and other small molecules were analyzed. This analysis allowed us to further compare our in silico evaluation of USP interactions with the results obtained in other studies.

To experimentally confirm the likelihood of LAT1 binding to tryptophan (Trp) and 3-amino-2-norbornanecarboxylic acid (BCH), the human LAT1–4F2hc complex was isolated from HEK293F cells by recombinant expression. Further purification of LAT1 and 4F2hc was carried out. The complex was reconstituted in liposomes and a countercurrent assay was performed, in which [^3^H]leucine substrate uptake by proteoliposomes was monitored. Experimental results showed that [^3^H]leucine transport was inhibited by large neutral amino acids, including Trp and BCH. The half-maximal inhibitory concentration (IC50) of these compounds for [^3^H]leucine transport inhibition was measured. LAT1 has been shown to have an inwardly open conformation, with BCH bound in close proximity to the Phe252 side chain and at the center of the putative transport pathway. LAT was shown to be involved in hydrophobic interactions with Phe252 benzene ring, while carboxyl and amino groups form hydrogen bonds with the atoms of TM1 and TM6 LAT1 main chains, respectively [151]. Based on structural analysis of sequences with other amino-acid antiporters, six potential gating residues on LAT1 were characterized: Tyr117, Phe252, Trp257, Asn258, Tyr259 and Arg348. All of them were located along the proposed transport path in the transporter [152]. Singh N. et al. (2018) evaluated 30 compounds for their ability to inhibit LAT1-mediated histidine transport in proteoliposomes, reconstituted with recombinant purified human LAT1. The complexes were measured at a concentration of 100 μM vs. 1 (BCH) as a positive control and dimethyl sulfoxide (DMSO) as a negative control. S-(3-bromo-4-methoxybenzyl)-l-cysteine (number **28**), l-2-amino-4-(3,5-dichlorophenyl)butanoic acid (number **36**) and 5-(benzyloxy)-tryptophan (number **42**) compounds were highly active. Compounds **36** and **42** showed complete inhibition of LAT1 with IC50 values of 0.64 ± 0.12 and 1.48 ± 0.27 μM, respectively, while compound **28** had an IC50 of 33.2 ± 4.5 μM. The amino-acid moiety was critical for LAT1 recognition. This observation was consistent with previous studies demonstrating that compounds lacking this chemical motif did not bind to LAT1 [153].

According to these data, amino-substituted compounds with a free α-carboxyl group did not inhibit LAT1 as well, which indicated the importance of the free α-amino group in this process [154]. The α-amino group seems to be of more importance than the α-carboxyl group. It is assumed that substitutions in the α-amino or α-carboxyl group are sterically prohibited, since they prevent the binding of the polar head group, which leads to low-affinity binding of a ligand (α-carboxyl substitution and free α-amino group) or complete binding failure of a ligand (α-amino substitution and free α-carboxyl group or substitutions in both groups). This hypothesis, supported by docking, is consistent with the CoMFA LAT1 model, which demonstrated that the steric features’ addition beyond the amino-acid terminus reduced affinity [155].

However, other studies have shown that an amino-acid moiety was not a strict requirement for binding to LAT1 and that it was possible to replace the alkoxyoxygen of the α-carboxyl group with a hydroxamic acid moiety or modify the α-carboxyl group to carboxylic esters [154,156]. In addition, the optimal position of the amino group was observed at the α-carbon atom. This observation was consistent with previous results demonstrating that the distance between positive and negative charges should not exceed ~3 Å. If the amino group was located far from the carboxyl group, then the compound lost its affinity to LAT1 [157]. In terms of rigidity, the conformational restriction induced by cyclohexane seems to be more favorable for LAT1 activity than cyclopentane or other carbocycles. In addition, hydrophobicity plays an important role in side chain binding [158]. LAT1 was found to be able to bind and transport ligands with polar and ionizable fragments substituted in the meta position of phenylalanine, which indicated the disordered nature of the binding site [159]. The docking geometry of all active connections corresponded to the expected CBM (common binding mode).

**Figure 1:**
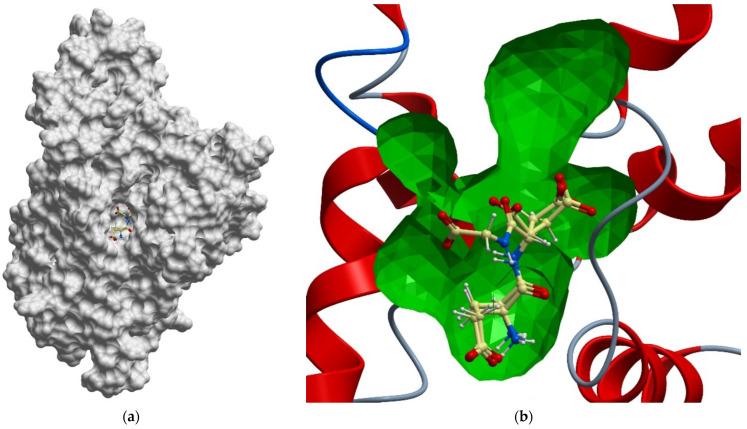
USP complex with LAT1 transporter obtained via molecular modeling and docking of mobile ligands: (**a**) view of the van der Waals surface of LAT1 transporter from the side of the ligand binding center; (**b**) close-up of the LAT1 binding site in complex with di- and tripeptide EG and EDG. The far side of the inner surface of the LAT1 ligand-binding site is shown in green.

The ligand-binding site of the LAT1 transporter is a deep cavity with a large number of charged, polar and hydrophobic groups, which open up many binding opportunities for ligands with different structures (Figure 1).

Visual analysis of the available spatial structures of LAT1 (PDB: 6IRT, 6IRS, 6JMQ, 7DSQ, 7DSN, 7DSL and 7DSK) [152,160] shows that the size of the active site and the amino-acid composition allows the binding of different USPs that are much larger than amino acids.

Using modern methods of virtual screening, computer docking of conformationally flexible compounds and drug design (ICM-Pro, Molsoft LLC), the binding efficiency of known LAT1 ligands (Table 1) with some biologically active USPs (Table 2) has been compared.

According to the calculations, native ligands and LAT1 inhibitors presented in Table 1 have an ICM-Score from −19.67 to −15.00, which corresponds to IC50 micromolar range values and a high probability of binding to the LAT1 transporter. Thus, ICM-Score function values of the studied USPs less than −15 may indicate a high probability of highly efficient binding to the LAT1 transporter.

Table 2 shows the docking results of 27 biologically active peptides in the NSP5 active site. It can be seen that the ICM-Score of 20 out of 27 USPs is no worse than that of the native ligands presented in Table 1. This means that di-, tri- and some tetrapeptides have both enough free space in the active center and the ability to saturate their hydrogen bonds, and find partners in electrostatic and hydrophobic interactions, which is indicative of the possibility for USP data to be transported across the membrane by LAT1 transporter.

## 9. Discussion

Due to their high biological activity and low immunogenicity, USPs are suitable for the pharmacotherapy of many pathological conditions. USPs are convenient for synthesis, are biocompatible, are accessible for chemical modifications, and demonstrate molecular selectivity and specific interaction with various types of biological systems [179,180,181].

Physiological properties of biologically active peptides imply their direct effect on target tissues and organs; that is, when administered orally, peptides must be absorbed into the bloodstream through the intestinal barrier in their active forms. It is important to study the absorption mechanism of bioactive peptides for a better understanding of their biological effects [182,183].

Di- and tripeptide transporters are integral proteins of the plasma membrane. PEPT1, PEPT2, PHT1, PHT2 transporters are members of the proton-coupled oligopeptide transporter (POT) family; LAT1, LAT2, LAT3, LAT4 constitute the L system. Apparently, these transporter systems provide extra- and intracellular transport of USPs consisting of 2–4 amino-acid residues. PEPT1 is expressed in the small intestine; PEPT2 is expressed mainly in the brain and kidneys, as well as in the gastrointestinal tract, liver and lungs during late embryogenesis. This suggests that PEPT1 may serve as a peptide transporter in the embryonic period. Tissue localization of PHT1 and PHT2 transporters is currently poorly understood, although it is known that they are expressed in the brain. PEPT1 is believed to provide transport of di- and tripeptides, as well as some peptidomimetics and prodrugs, through the apical membrane of enterocytes, where they are hydrolyzed. After that, other amino acid and/or peptide transporters (probably LAT) deliver these substances into the blood. In kidneys, PEPT2 is responsible for reabsorption of these substances. Dipeptides, tripeptides, beta-lactams, ACE inhibitors and prodrugs are absorbed by cells against a concentration gradient by PEPT1 and PEPT2 carriers, whose activity is associated with an electrochemical proton gradient.

Some di- and tripeptides undergo a rapid intracellular hydrolysis, after which amino acids are transported out of the cell via basolateral transporters. Hydrolysis-resistant substrates, which include most peptidomimetics, are released into the blood via a yet-to-be-identified basolateral peptide carrier and/or via other drug-transport systems. It is very important that not all USPs undergo a rapid intracellular hydrolysis [184,185]. It has been established that the dipeptides formed during the hydrolysis of the N-terminal fragments of the polypeptide chain by Asp, Gly and Pro or the hydrolysis of the C-terminal fragments by Pro, Ser, Thr and Asp are slightly hydrolysable. Their stability and biological activity have been confirmed experimentally [182]. A study on the hydrolysis of AEDG, KE, KEDW, KEDA peptides in saline, Ringtger’s solution, HCl in stomach homogenates, small and large intestines, and other organs (liver, kidneys and spleen) has been conducted. Tissue homogenate hydrolysis was assessed by cleavage of the terminal amino acid from each peptide using L-aminopeptidase. KEDW and KEDA peptides were found to be resistant to hydrolysis for 3–4 h in physiological saline, Ringtger’s solution, HCl and tissue homogenates [186]. KE peptide (to a greater extent) and AEDG peptide (to a lesser extent) were hydrolyzed in solutions and partially in tissues. For AEDG peptide, it remains unclear whether only the terminal amino acid was cleaved, or whether the remaining tripeptide was cleaved as well. These data may indicate a different degree of USP hydrolyzability and the presence of hydrolysis-resistant ones among them.

Competitive inhibition of PEPT1/2, regulation of PEPT1/2 transcription and maintenance of the proton gradient are the factors for the regulation of transport via these carriers [186,187].

LAT1 and LAT2 carriers are involved in the pathogenesis of some diseases. In particular, their role in the tumor onset and development is being actively studied. LAT1 expression has been described as an important indicator of poor outcome in various human cancers [126,188,189,190].

The role of LAT1 and LAT2 amino-acid carriers in drug transport is being intensively studied. LAT1 preferentially transports large neutral amino acids, while LAT2 exhibits a broader substrate specificity, also transporting some smaller neutral amino acids. The exact localization of LAT1 and LAT2 on cell membranes is not always clear, while data on their distribution in tissues is sometimes contradictory. LAT1 is expressed in many tissues under normal conditions, as well as in a number of tumors. LAT2 is expressed in the kidney, colon and intestine. The potential effect of LAT1 and LAT2 on pharmacokinetics is limited by passive diffusion, competitive inhibition due to high levels of endogenous amino acids and relatively low drug affinity to LAT proteins. Some calculations show that it is not drug substrates (such as L-DOPA and metphalan) that predominate as a substrate in the LAT system, but amino acids, which is why LAT-mediated drug transport through the BBB is unlikely [16]. In addition to amino-acid transport, LAT1 is involved in the transport of thyroid hormones—triiodothyronine and thyroxine [191].

**Figure 2:**
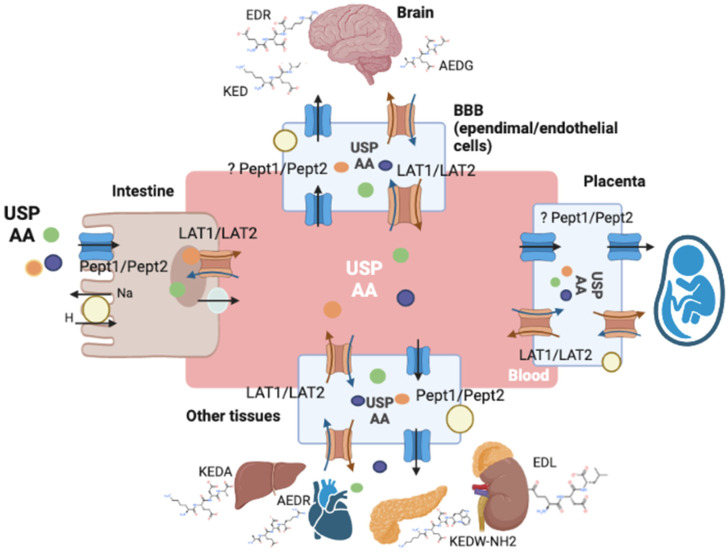
Schematic representation of the short-peptide transport via carrier proteins.

The results of molecular modeling (Table 1) confirmed that some amino acids and their chemical modifications had a high probability of binding to the LAT1 transporter. In addition, the calculation for USPs showed that of 27 peptides, possessing different biological activity and presented in Table 2, 20 had a high degree of affinity to the LAT1 transporter. The highest affinity to LAT1 was found for the following peptides: ED (genitourinary system regulator), EDG (gastroprotector) and EDR (neuroprotector). Of the 20 peptides showing a high probability of binding to LAT1, 4 are neuroprotectors, 4 are urinary system regulators, 3 are cardioprotectors, 2 are immunoprotectors, 2 are hepatoprotectors, 1 is a gastroprotector, 1 is a nephroprotector, 1 is a chondroprotector, 1 is a bronchoprotector and 1 is involved in the endocrine regulation. Some peptides, such as KED, possess multiple biological activities. According to the literature data, LAT1 is expressed ubiquitously [192], the highest expression levels are observed in the kidneys and bladder, gastrointestinal tract, brain, testicles, placenta, heart muscle and bone marrow. Thus, the tissue-specific effects of some USP can be explained by their high affinity to the LAT1 transporter expressed in target tissues. However, not all USPs studied showed a high probability of binding to LAT1 while nevertheless showing tissue-specific activity. In particular, LAT1 is moderately expressed in the prostate, but KEDP and AE peptides, responsible for prostatic function regulation, do not demonstrate a high binding ability to LAT1 [193]. It is likely that LAT1 partially explains the mechanism of USP penetration into tissues, but to get a more thorough understanding, additional studies using molecular modeling methods are needed in order to identify the features of the USP interaction with other L and POT system carriers. Figure 2 gives a schematic representation of the short-peptide transport through carrier proteins in various tissues. Orally ingested amino acids and USPs are transported to intestinal cells, presumably by the PEPT1 transporter at the apical side of the membrane, and afterwards they enter the blood flow through the basolateral membrane via L-system transporters. BBB, placenta and a number of other body tissues express L-system carriers as well as PEPT1 and PEPT2 carriers, which provide transport from the blood into the cell and from the cell to the target tissue. A high level of LAT1 expression in a number of tissues implies that this particular transporter provides peptide penetration into target tissues, but a few studies on LAT2, as well as PEPT1 and PEPT2, imply their involvement in the transport of USP and amino acids.

## 10. Conclusions

Molecular modeling data covering 20 biologically active di-, tri- and tetrapeptides and literature analysis allow us to conclude that there is a high probability of their transport into cells of various organs and tissues with the participation of LAT1 transporter, POT family and L-system transporters. Further study of the USP cell transport is important for understanding the molecular mechanisms of their biological activity and development of targeted drugs on their basis.

## Figures and Tables

**Table 1:** Binding of various ligands to LAT1, human L-type neutral amino-acid transporter.

Ligand Structure	Mol. Name	ICM-Score	IC50, µM	Reference
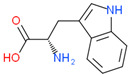	Tryptophan	−19.67	20.16	[151]
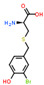	S-(3-bromo-4-methoxybenzyl)-l-cysteine	−19.31	33.20	[152]
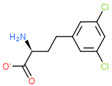	l-2-amino-4-(3,5-dichlorophenyl)butanoic acid	−17.56	0.64	[152]
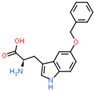	5-(benzyloxy)-tryptophan	−17.59	1.48	[152]
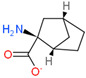	2-Amino-2-norbornanecarboxylic acid (BCH)	−15.00	155.10	[152]

**Table 2:** Binding of ultrashort peptides to LAT1, human L-type neutral amino-acid transporter.

2D Structure of Peptide	Name and Structure of Peptide	ICM-Score	Biological Activity
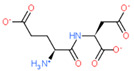	Vesilut (ED)	−34.32	Bladder regulator
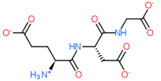	Chonluten (EDG)	−30.30	Gastroprotector [161] Stress protector [162]
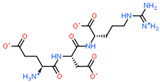	Pinealon (EDR)	−30.29	Neuroprotector [163]
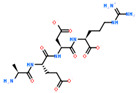	Cardiogen (AEDR)	−29.81	Cardioprotector [164]
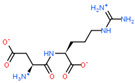	Prostataget (DR)	−28.88	Regulation of prostatic functions
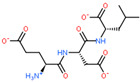	Ovagen (EDL)	−28.27	Nephroprotector [165] Hepatoprotector [166]
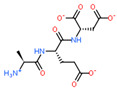	Cartalax (AED)	−26.65	Chondroprotector [167] Regulation of skin fibroblast functions [168]
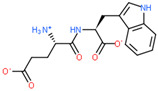	Thymogen (EW)	−24.72	Immunoprotector, medicinal product [14,169]
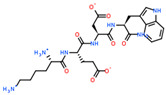	Pancragen (KEDW-NH2)	−23.47	Regulator of pancreatic functions [170]
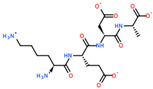	Livagen (KEDA)	−22.01	Hepatoprotector [171]
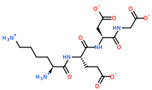	Testagen (KEDG)	−21.92	Regulator of the male reproductive system
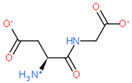	Kistiget (DG)	−21.03	Bladder regulator
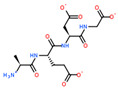	Epitalon (AEDG)	−20.38	Geroprotector [12] Regulator of the epiphysis functions [172] Neuroprotector [173]
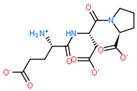	Crystagen (EDP)	−20.22	Immunoprotector [174]
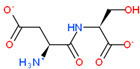	Vesselget (DS)	−19.82	Vasoprotector [174]
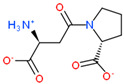	Nefroget (H-Asp(Pro)-OH)	−19.66	Nephroprotector
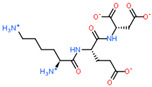	Vesugen (KED)	−18.91	Vasoprotector [13] Neuroprotector [175] Geroprotector [176]
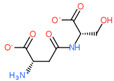	Subcortiget (H-Asp(Ser)-OH)	−18.78	Neuroprotector
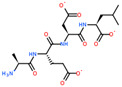	Bronchogen (AEDL)	−17.26	Bronchoprotector [170]
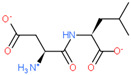	Fuaget (DL)	−16.01	Hepatoprotector
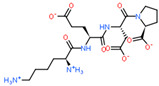	Prostomax (KEDP)	−13.58	Regulation of prostatic functions [177]
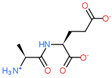	Orchiget (AE)	−13.03	Regulation of prostatic functions
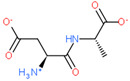	Retiget (DA)	−12.82	Retinaprotector
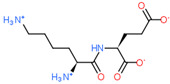	Vilon (KE)	−12.31	Immunoprotector [14,170]
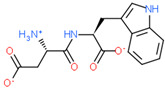	Thyroidget (DW)	−12.12	Thyroid regulator
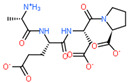	Korthagen (AEDP)	−12.11	Neuroprotector [13,170]
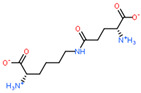	Normoftal (H-Lys(H-Glu-OH)-OH)	−10.49	Retinaprotector [178] Immunoprotector [170]

## Data Availability

Not applicable.
